# Obesity- and Lipid-Related Parameters in the Identification of Older Adults with a High Risk of Prediabetes According to the American Diabetes Association: An Analysis of the 2015 Health, Well-Being, and Aging Study

**DOI:** 10.3390/nu11112654

**Published:** 2019-11-04

**Authors:** Robinson Ramírez-Vélez, Miguel Ángel Pérez-Sousa, Katherine González-Ruíz, Carlos A. Cano-Gutierrez, Jacqueline Schmidt-RioValle, María Correa-Rodríguez, Mikel Izquierdo, Jesús Astolfo Romero-García, Adriana Yolanda Campos-Rodríguez, Héctor Reynaldo Triana-Reina, Emilio González-Jiménez

**Affiliations:** 1 Department of Health Sciences, Public University of Navarra, Navarrabiomed-Universidad Pública de Navarra (UPNA)—Complejo Hospitalario de Navarra (CHN), Instituto de Investigación Sanitaria de Navarra (IdiSNA), 31008 Pamplona, Navarra, Spain; mikel.izquierdo@gmail.com; 2 Centro de Investigación Biomédica en Red de Fragilidad y Envejecimiento Saludable (CIBERFES), Instituto de Salud Carlos III, 28029 Madrid, Spain; 3 Faculty of Sport Sciences, University of Huelva, Avenida de las Fuerzas Armadas s/n, 21007 Huelva, Spain; perezsousa@gmail.com; 4 Grupo de Ejercicio Físico y Deportes, Vicerrectoría de Investigaciones, Universidad Manuela Beltrán, Bogotá, DC 110231, Colombia; katherine.gonzalez@docentes.umb.edu.co; 5 Hospital Universitario San Ignacio—Aging Institute, Pontificia Universidad Javeriana, 110111 Bogotá, Colombia; ccano@javeriana.edu.co; 6 Department of Nursing, University of Granada, Av. Ilustración, 60, 18016 Granada, Spain; jschmidt@ugr.es (J.S.-R.); macoro@ugr.es (M.C.-R.); emigoji@ugr.es (E.G.-J.); 7 GICAEDS Group, Faculty of Physical Culture, Sport and Recreation, Universidad Santo Tomás, Bogotá 110311, Colombia; astolforomero@usantotomas.edu.co (J.A.R.-G.); adrianacampos@usantotomas.edu.co (A.Y.C.-R.); hectortriana@usantotomas.edu.co (H.R.T.-R.)

**Keywords:** adiposity, prediabetes, lipids, anthropometric measure, elderly

## Abstract

This study evaluated the predictive ability of 11 obesity- and lipid-related parameters, including body mass index (BMI), waist circumference (WC), waist-to-height ratio (WtHR), body roundness index (BRI), “A” body-shape index (ABSI), conicity index (C), visceral adiposity index (VAI), triglyceride-to-glucose fasting index (TyG), triglyceride-to-glucose fasting related to BMI (TyG-BMI), triglyceride-to-glucose fasting related to WC (TyG-WC), and triglyceride-to-glucose fasting related to WtHR (TyG-WtHR), to identify patients from an elderly Colombian population with a high risk of prediabetes according to the 2016 American Diabetes Association criteria. The data were obtained from the 2015 Colombian Health and Wellbeing and Aging Survey. A total of 3307 elderly Colombian individuals (aged over 60 years) were included. Anthropometric data, fasting plasma glucose, blood lipid profiles, family history, and health-related behaviors were assessed, and prediabetes was defined as a fasting plasma glucose of 100 to 125 mg/dL. The areas under the receiver operating characteristic (ROC) curves (AUCs) were calculated for each anthropometric indicator, using the prediabetes classification to identify their sensitivity and specificity, and these indicated that the prevalence of prediabetes was 25.3% in this population. After adjusting for potential confounding factors, the TyG index was strongly associated with the odds of having prediabetes in both sexes, and multivariate logistic regression analysis showed that the ORs for prediabetes increased across quartiles (*p* < 0.001). The TyG index was best able to identify prediabetes in either sex (AUC and optimal cut-off = 0.700 and 8.72, and 0.695 and 8.92 for men and women, respectively), suggesting that compared to the other parameters, the TyG index has the best discriminative power to predict prediabetes in the whole population. Thus, we propose the TyG index be used as a complementary marker for assessing prediabetes in older adults.

## 1. Introduction

Prediabetes is a complex, multifactorial metabolic disorder that extends beyond glucose control. The presence of prediabetes increases the risk of developing type 2 diabetes mellitus (T2DM) 3-fold to 10-fold [1]. In addition, previous studies have reported that there are cause–effect relationships between prediabetes and cardiovascular disease such as coronary heart disease or stroke, and all-cause mortality [2,3,4,5]. According to the World Health Organization, and depending on the diagnostic criteria used (e.g., age, ethnicity, sex, etc.), the prevalence of fasting hyperglycemia in the Americas region in 2014 was 8.1% in women and 9.3% in men [6]. The International Diabetes Federation projects that the global prevalence of prediabetes will increase to reach approximately 471 million people by 2035, with an increase of between 6.9% and 8.0% between 2013 and 2035 [7]. This metabolic disorder is usually identified by dysglycemia, with impaired fasting glucose (IFG; defined as a fasting plasma glucose of 100 to 125 mg/dL) and/or impaired glucose tolerance (IGT, defined as a plasma glucose level of 140–200 mg/dL 2 h after ingesting a 75 g oral glucose load) [7].

Notably, no single definition for prediabetes has yet been universally accepted by either the research or the public health community [8]. This has led to major differences in the numbers of individuals classified as high-risk prediabetics in different countries, and hinders determinations of who should be fast-tracked into prevention programs [9]. In a meta-analysis evaluating the progression of prediabetes to T2DM published by Gerstein et al. [10], the annual incidence of T2DM was 4–6% for IGT, 6–9% for IFG, and 15–19% for when considering both IGT and IFG together. This theory supports the use of dysglycemia as a valuable and widely used method to measure the progression of prediabetes to T2DM.

However, because diagnosing prediabetes requires invasive laboratory tests to determine the plasma glycemic, glycated hemoglobin, and fasting plasma insulin levels, its inclusion on a large scale in the routine monitoring of the health status of elderly people is complex. Thus, research is being directed towards identifying more affordable alternatives for epidemiological tracking so that specific procedures can be targeted towards the patients most at risk of developing prediabetes. In this context, numerous studies have suggested simple surrogate obesity- and lipid-related indices for identifying metabolic disorders, including the BMI, WC, WtHR, BRI, ABSI, and C [11,12,13]. Additionally, other indices such as the VAI, TyG index, TyG-BMI, TyG-WC, and TyG-WtHR have been widely used in epidemiological research because they are easy and practical to apply and are more efficient than previously used markers [9,11,12,13].

While available guidelines for T2DM prevention and treatment show that both old age (60 years or more) and prediabetes are important risk factors for diabetes [9,13], a comprehensive consensus has yet not been reached on the best indices for evaluating the status and risk of prediabetes in the elderly Latino population. Thus, considering that prediabetes patients aged over 60 years have a very high probability of developing diabetes, we decided to investigate the clinical utility of several surrogate clinical markers for the detection of this pathology in this population. According to the PubMed database (https://www.ncbi.nlm.nih.gov/pubmed) no study has yet compared all 11 obesity- and lipid-related indices side by side as prediabetes predictors, meaning that the arguments continue about which parameter best conveys the risk of T2DM.

Dysglycemia is a key component in metabolic syndrome and insulin resistance, and therefore, obesity- and lipid-related indices might help to identify prediabetes. Because weight gain and body-fat distribution changes significantly increase the risk of insulin resistance in older adults, it is important to identify which patients are at a high risk of developing T2DM so that prevention and intervention strategies can be implemented in a timely manner. Thus, in this study, we investigated the clinical utility of 11 surrogate clinical markers for the detection of prediabetes in elderly Colombian patients.

## 2. Materials and Methods

### 2.1. Study Population

The data for this secondary cross-sectional study were obtained from the 2015 Colombian Health and Wellbeing and Aging Survey (SABE, from the Spanish initialism Salud, Bienestar & Envejecimiento, 2015), a multicenter project conducted from 2014 to 2015 by the Pan-American Health Organization and supported by the Epidemiological Office of the Ministry of Health and Social Protection of Colombia (https://www.minsalud.gov.co/). All the participants were randomly selected, voluntarily enrolled in the survey, and provided their informed consent to participation. This survey was reviewed and approved by the institutional review boards at the University of Caldas (ID protocol CBCS-021-14) and the University of Valle (ID protocol 09-014 and O11-015). The study protocol for the secondary analysis was approved by the Human Subjects Committee at Pontificia Universidad Javeriana (ID protocol 20/2017-2017/180, FM-CIE-0459-17) in accordance with the World Medical Association Declaration of Helsinki and Resolution 8430 for the technical, scientific, and administrative standards for conducting research with humans, published in 1993 by the former Colombian Ministry of Health.

The SABE included individuals in the Colombian population aged over 60 years, and the indicators were disaggregated by their age ranges, sex, ethnicity, and socioeconomic status, details of which have been previously published [14]. Most of the population included in the study (99%) resided in private homes in the urban and rural stratification of the sample, and the sample was selected in segments according to the municipal cartography of the area (as published by the Epidemiological Office of the Ministry of Health and Social Protection of Colombia). A total of 23,694 surveys were conducted nationally, in a total of 6365 population segments located in 246 municipalities. There were an average of 4.2 ± 1.2 adults per segment, and the means and proportions for the SABE were estimated with a degree of error of up to 6% of the maximum expected error at the national disaggregation level only.

**Figure 1:**
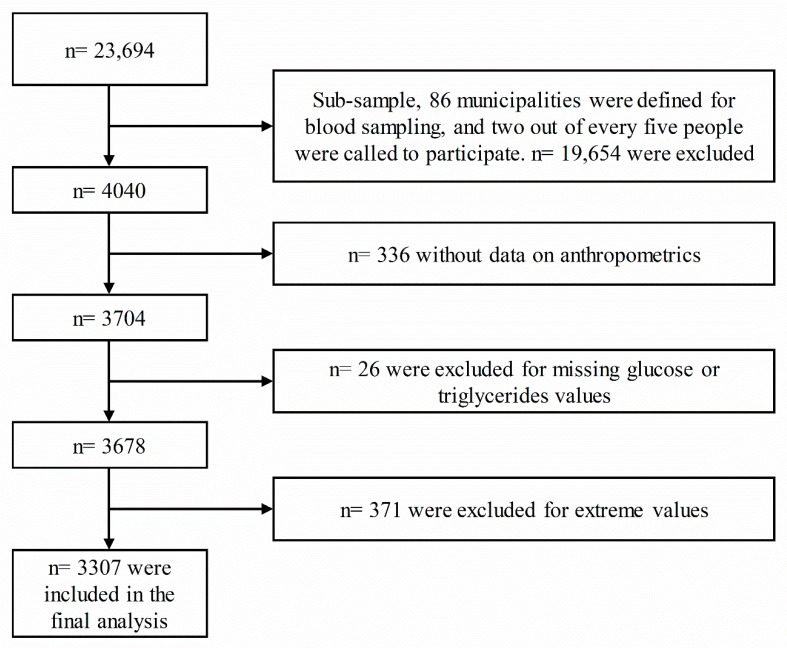
Flow chart showing the selection of the study sample from the Colombian Health and Wellbeing and Aging Survey (SABE) 2015. All analyses presented in this paper were based on 3307 surveyed participants, each with complete anthropometric, blood-based indicator, and covariable data.

The participants were systematically selected according to the sampling fraction with respect to the general SABE sample. The following participants were excluded: patients with type 1 diabetes; patients who had previously received treatment with antidiabetic drugs or who were hyperlipidemic; participants with missing demographic, anthropometric, or laboratory data values; and those who had not fasted for at least 8 h before testing. Visual inspection of the data using boxplots revealed 371 outliers (determined using the interquartile rule); the population mean BMI was 50 kg/m^2^, mean triglycerides were 500 mg/dL, HDL was 100 mg/dL, and WC was 130 cm. In this subsample, 86 municipalities were defined for blood sampling and two out of every five people were called to participate. A total of 3307 patients were finally included in our analysis, as described in the flow chart diagram shown in Figure 1.

### 2.2. Anthropometric Measurements

Physical examinations were performed by trained staff according to a standardized protocol [15]. Body weight and height were measured with the patient wearing light indoor clothing using a Kendall graduated platform scale and a SECA 213^®^ stadiometer (Hamburg, Germany), and BMI was calculated using the formula BMI = weight (kg)/height (m^2^). WC was measured midway between the costal margin and the iliac crest at the end of a normal expiration, and WHtR was calculated using the formula WHtR = WC (cm)/height (cm). The remaining anthropometric indices, including BRI, BAI, ABSI, C, VAI, TyG index, TyG-BMI, TyG-WC, and TyG-WHtR, were calculated using the following Equations [16,17,18,19,20,21,22,23]:

– BRI = 364.2 − 365.5 [1 − π^−2^ WC^2^ (m) Height^−2^ (m)]^1/2^
– BAI = [Hip circumference (m)/Height^2/3^ (m)] − 18
– ABSI = WC (m)/[BMI^2/3^(kg/m^2^)Height^1/2^ (m)]
– C = 0.109^−1^ WC (m) [Weight (kg)/Height (m)]^−1/2^
– VAI = Males: [WC/39.68 + (1.88 × BMI)] × (TG/1.03) × (1.31/HDL); Females: [WC/36.58+(1.89 × BMI)] × (TG/0.81) × (1.52/HDL)
– TyG index = Ln[(triglyceride (mg/dl) × glucose (mg/dl)/2]
– TyG-BMI = TyG × BMI
– TyG-WC = TyG × WC
– TyG-WHtR = TyG × WHtR

### 2.3. Laboratory Measurements

After an overnight fast, blood was collected in the morning. The blood samples were centrifuged for 10 min at 3000 rpm 30 min after sampling. All samples were delivered to a single central laboratory (Dinamica Laboratories, Bogotá, Colombia) for analysis within 24 h. Fasting plasma glucose and plasma triglycerides were analyzed through enzymatic colorimetric methods.

### 2.4. Classification of Variables

The diagnostic criterion for prediabetes based on IFG was defined as a fasting plasma glucose level of 100 to 125 mg/dL, according to the recent guidelines published by American Diabetes Association (ADA) in 2016 [24]. Self-reported questionnaires were used to determine smoking status (categorized as “never/previous smokers” as no smokers, and “current smokers” as yes), alcohol consumption during the past month (categorized as “no alcohol intake”, and “alcohol consumed less than once per week”, or “alcohol consumed two to six days per week, or everyday” as alcohol intake/yes), and exercise habits. For the latter, a “proxy physical activity” report was conducted using the following questions: (i) “Have you regularly exercised, (e.g., engaged in jogging or dancing activities, or performed rigorous physical activity at least three times a week for the past year?”; (ii) “Do you walk between 9 and 20 blocks (1.6 km) without resting at least three times a week?”; and (iii) “Do you walk 8 blocks (0.5 km) without resting at least three times a week?”. The participants were considered physically active if they responded affirmatively to two of the three questions. Medical information, including multimorbidity and chronic conditions adapted from the original SABE study, were assessed by asking the participants if they had been diagnosed with hypertension, respiratory disease, cardiovascular diseases (including heart attack or angina), stroke, osteoporosis, cancer, or sensory impairments (vision and hearing loss) by a physician.

### 2.5. Analysis Plan

All data were analyzed using SPSS version 24.0 for Macintosh (IBM Corp., Armonk, NY, USA) and MedCalc Statistical Software version 18.2 (MedCalc Software BVBA, Ostend, Belgium). The data are presented as the mean ± standard deviation (*SD*) for continuous variables and as frequencies and percentages for categorical variables. The normality of the variables was verified using Kolmogorov–Smirnov tests and probability plots. Student’s *t*-tests or Mann–Whitney U analyses were applied to identify significant differences in continuous variables and chi-squared tests were used for categorical variables. Gardner–Altman plots were produced using estimation statistics for data visualization [25], and point estimates were estimated using within-group unadjusted means and 95% confidence intervals (CIs). Significant within-group changes were indicated when the upper or lower limits of the 95% CIs did not cross zero.

The odds ratio (ORs) and 95% CIs of quartiles 2–4 for each surrogate index and their relationship with prediabetes were calculated and compared with quartile 1 as a reference group, adjusting for potentially confounding variables such as age, smoking, alcohol consumption, and physical activity habits. The ROC curves were plotted and the AUCs were calculated to examine the ability of these indices to identify prediabetes. Cut-off points were chosen based on the Youden index (sensitivity + specificity − 1) [26], which uses the point on the ROC parameter farthest from the line of equality.

The likelihood ratios (LR+ and LR−) were also determined, and the effect size (ES) and ORs of each index were calculated to determine the predictive magnitude of each metric [26]. The magnitude was interpreted by classifying them as “trivial” (<0.20), “small” (0.20 to <0.50), “moderate” (0.50 to <0.80), or “large” (≥0.80) values [27]. Finally, Chi-squared tests were used to determine differences in the prevalence of prediabetes based on the cut-off points, applying Cramer’s V to find the interpreted effect size based on the McHugh guidelines [28].

## 3. Results

### 3.1. Clinical and Sociodemographic Characteristics of the Study Participants According to Their Glycemic Status

The characteristics of the SABE study population, stratified by their glycemic status, are shown in Table 1. Of the 3307 participants, 1930 were women (58.3%) and 1377 were men (41.6%), and their combined mean age was 70.3 years. According to the ADA criteria, 839 (25.3%) individuals were prediabetic and 2468 were healthy. There were significant differences (*p* < 0.05) in the anthropometric characteristics between healthy and prediabetic patients in terms of their BMI (26.3 kg/m^2^ vs. 28.1 kg/m^2^, respectively), WC (91.1 cm vs. 95.4 cm, respectively), and WtHR (0.58 vs. 0.60, respectively). Likewise, triglycerides and glucose were significantly higher in prediabetic versus healthy patients.

The same significant differences between groups were also detected in the surrogate indices, except for the ABSI index. The prevalence of overweight and obese individuals in the overall sample was 39.3% and 26.7%, respectively. There were also significant differences in the distribution of all the weight status groups between prediabetic and healthy individuals. Regarding covariates such as smoking status and alcohol intake, most participants did not smoke (89.8%) or drink alcohol (87.4%); however, both groups tended to be physically inactive. Moreover, the most common comorbidities in the overall sample were hypertension (30.9%), vision loss (27.8%), osteoporosis (12.0%), cardiovascular diseases (9.4%), and hearing loss (8.2%).

### 3.2. Obesity- and Lipid-Related Parameters According to the 2016 American Diabetes Association Glycemic Status

**Figure 2:**
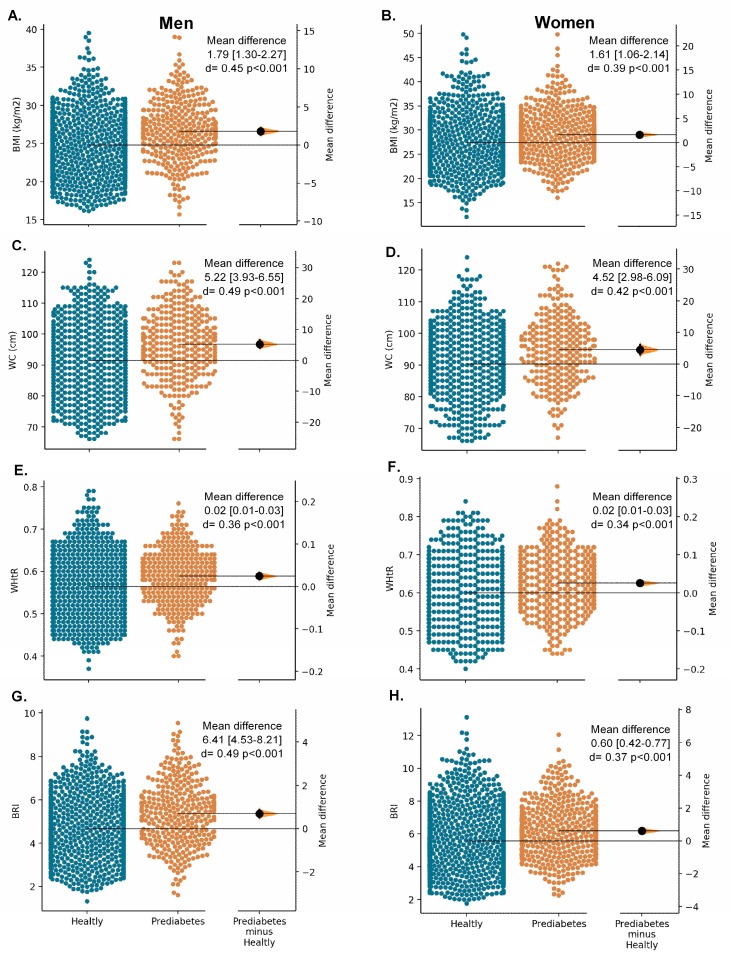
The Gardner–Altman plots for unadjusted surrogate obesity indices (BMI, WC, WHtR and BRI) according glycaemia status and sex.

**Figure 3:**
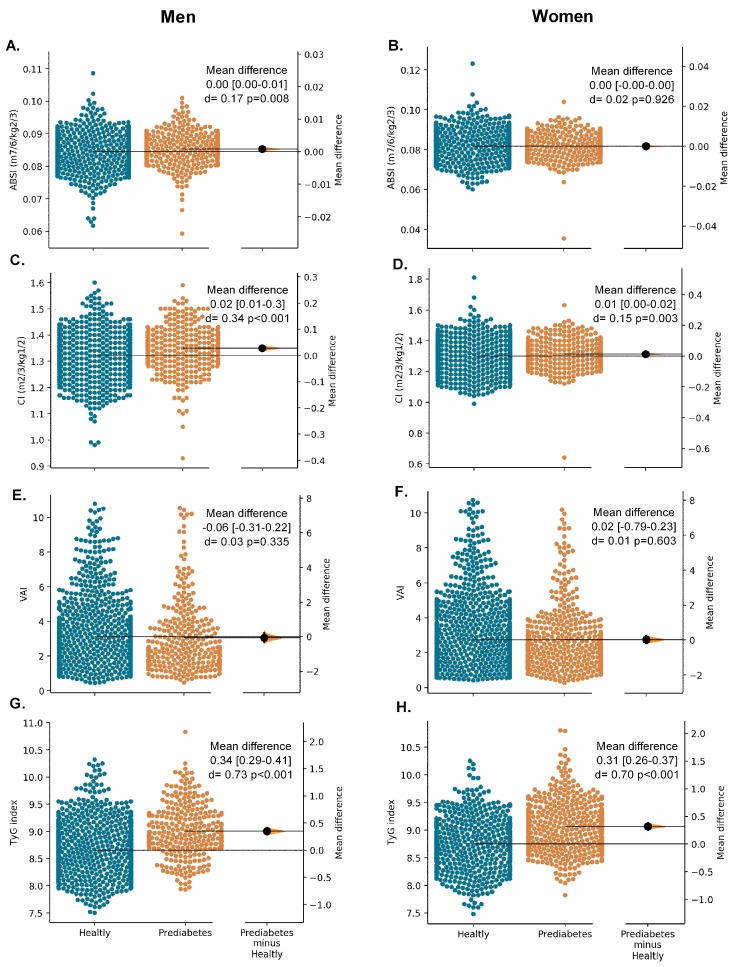
The Gardner–Altman plots for unadjusted surrogate obesity indices (ABSI, C, VAI, and TYG) according to glycaemia status and sex.

**Figure 4:**
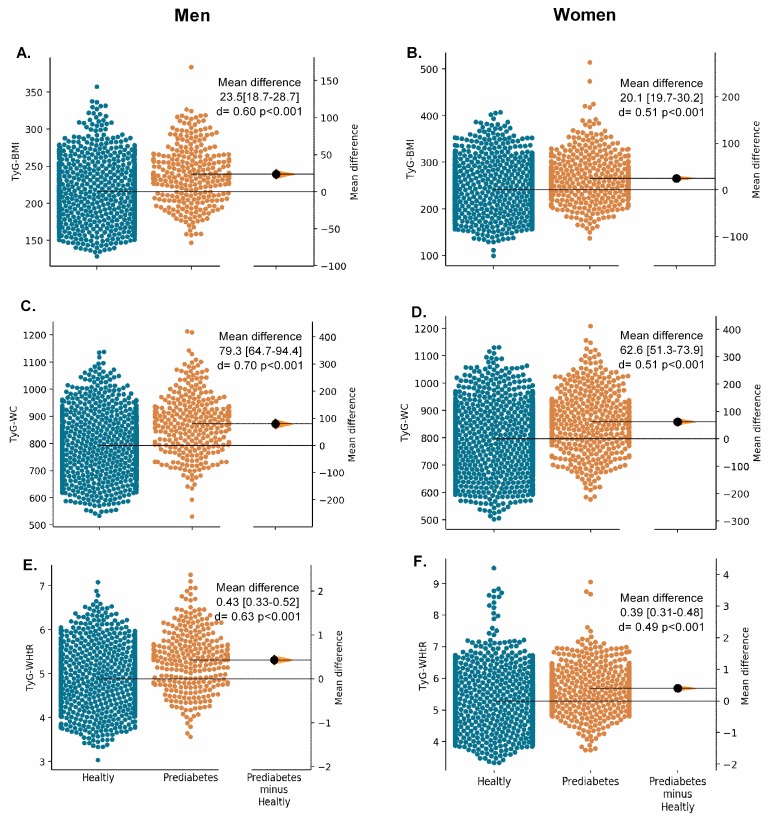
The Gardner–Altman plots for unadjusted surrogate obesity indices (TyG-BMI, TyG-WC, and TyG-WHtR) according to glycaemia status and sex.

Figure 2, Figure 3 and Figure 4 show the Gardner–Altman plots for the SABE study population obesity- and lipid-related parameters stratified by their glycemic status. We found significant differences in the glycemic status for all measurements except for ABSI in women (*p* = 0.926) and VAI in both sexes, Figure 3 (*p* > 0.05).

### 3.3. Association of Prediabetes with the Level of Obesity- and Lipid-Related Indices

**Figure 5:**
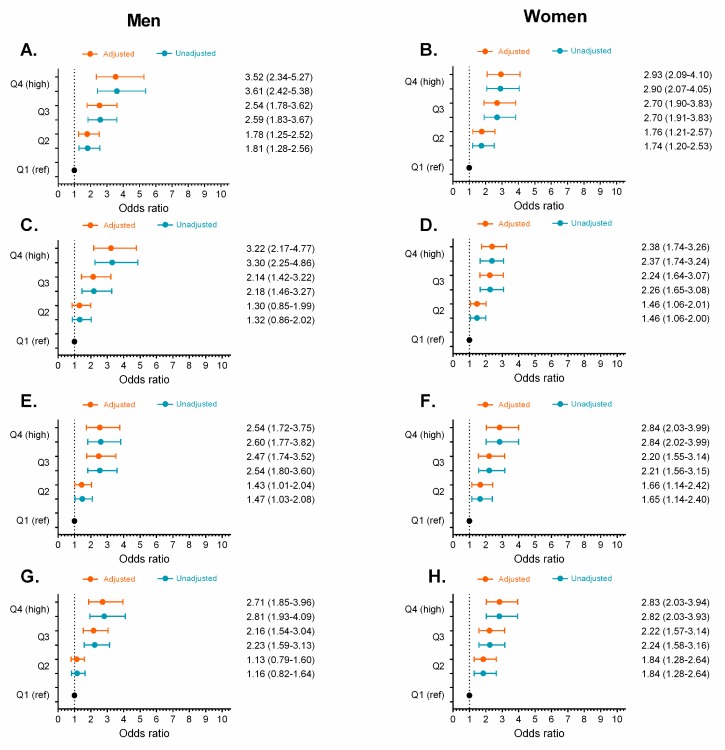
Unadjusted odds ratios (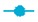) and adjusted odds ratios (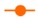) for prediabetes in quartiles (Q) of obesity- and lipid-related indices by sex. BMI (**A,B**), WC (**C,D**), WHtR (**E,F**), and BRI (**G,H**) by sex. Odds ratio adjusted for age, smoking, drinking, and physical activity “proxy”. (Q1 reference “lowest” group), second quartile (Q2), third quartile (Q3), and fourth quartile (Q4 highest group).

**Figure 6:**
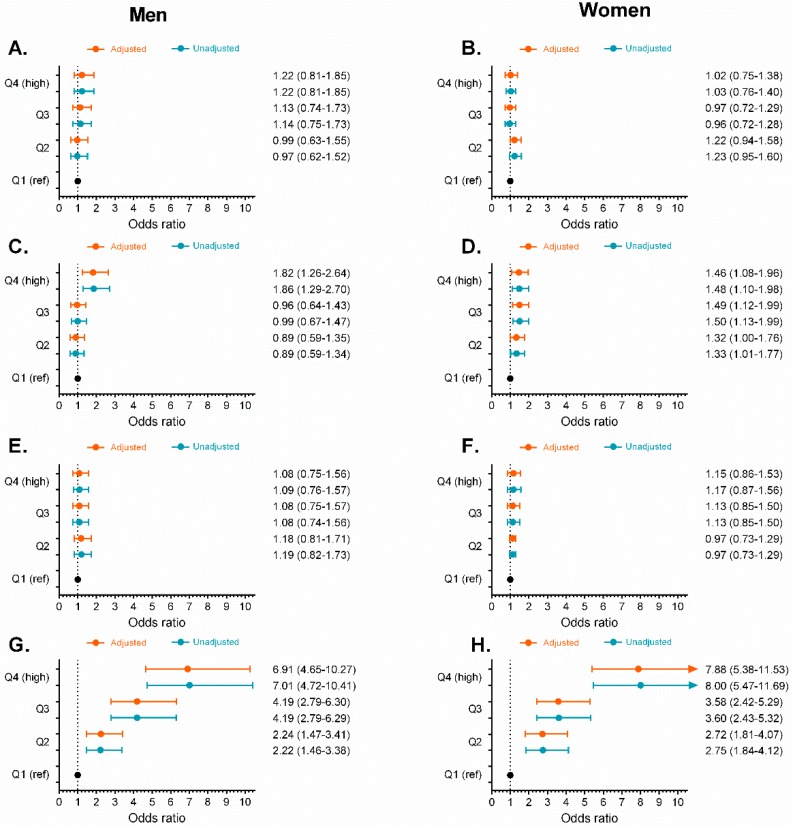
Unadjusted odds ratios (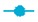) and adjusted odds ratios (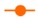) for prediabetes in quartiles (Q) of obesity- and lipid-related indices by sex. ABSI Panel (**A**,**B**), C (**C**,**D**), VAI (**E**,**F**), and TyG (**G**,**H**) by sex. Odds ratio adjusted for age, smoking, drinking, and physical activity “proxy”. (Q1 reference “lowest” group), second quartile (Q2), third quartile (Q3), and fourth quartile (Q4 highest group).

**Figure 7:**
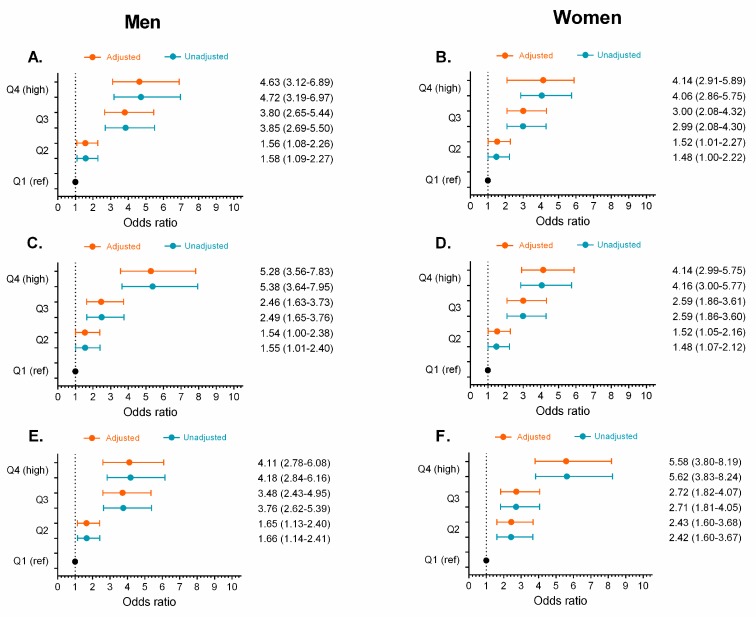
Unadjusted odds ratios (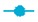) and adjusted odds ratios (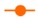) for prediabetes in quartiles (Q) of obesity- and lipid-related indices by sex. TyG-BMI Panel (**A**,**B**), TyG-WC (**C**,**D**), TyG-WHtR (**E**,**F**) by sex. Odds ratio adjusted for age, smoking, drinking, and physical activity “proxy”. (Q1 reference “lowest” group), second quartile (Q2), third quartile (Q3), and fourth quartile (Q4 highest group).

**Figure 8:**
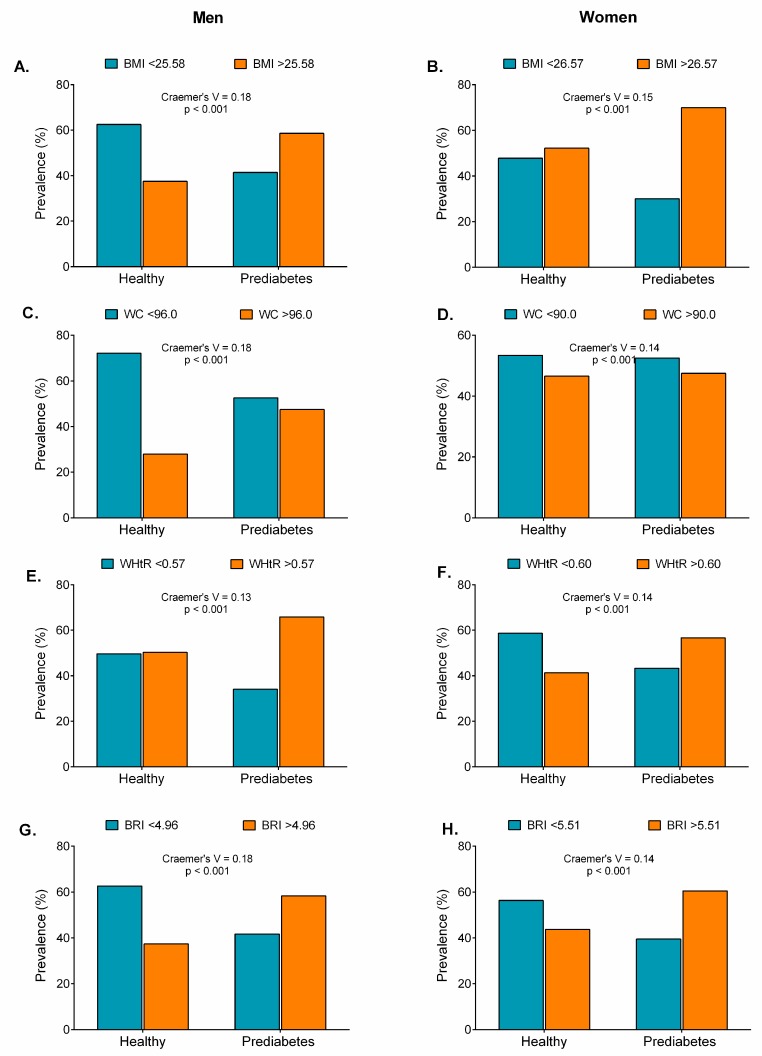
Prevalence of prediabetes according to obesity- and lipid-related index cut-offs (BMI, WC, WHtR, and BRI).

We divided each obesity- and lipid-related index indicator into increasing sex-specific quartile values and used logistic regression analysis to calculate the ORs and 95% CIs for prediabetes across the quartiles, using quartile 1 as a reference group, both without adjustments and after adjusting for age, smoking, alcohol consumption, and physical activity habits (see Figure 5, Figure 6, Figure 7 and Figure 8). The ORs for prediabetes generally increased in accordance with the increasing quartiles of each variable (*p* < 0.05), except for the ABSI and VAI indices (Figure 6).

In men, after adjusting for covariates, the ORs and 95% CIs for prediabetes were highest for the TyG index at 6.91 (95% CI [4.65, 10.27]) for the fourth quartile, compared to 2.24 (95% CI [1.47, 3.41], *p* < 0.001) for the second quartile. This was followed by TyG-WC (Q4 = 5.28; 95% CI [3.56, 7.83], *p* < 0.001) and TyG-BMI (Q4 = 4.63; 95% CI [3.12, 6.89], *p* < 0.001), as shown in Figure 6G and Figure 7C,E, respectively.

After adjusting for covariates in women, the ORs and 95% CIs were highest for prediabetes for the TyG index at 7.88 (95% CI [5.38, 11.53]) for the fourth quartile, compared to 2.72 (95% CI [1.81, 4.07], *p* < 0.001) for the second quartile, followed by TyG-WC (Q4 = 4.14; 95% CI [2.99, 5.75], *p* < 0.001) and TyG-BMI (Q4 = 4.14; 95% CI [2.91, 5.89], *p* < 0.001), as shown in Figure 6H and Figure 7B,D. In contrast, the ORs (*p* > 0.05) were lowest for the ABSI, C and VAI (Figure 6B,F) and the ORs were not statistically significant for the ABSI and VAI at any of their quartiles (Figure 6B,F).

### 3.4. Receiver Operating Characteristic Curve Analysis for the Obesity- and Lipid-Related Indices for Diagnosing Prediabetes According the 2016 American Diabetes Association Criteria

Results from the ROC analysis and AUCs for the 11 indices are shown in Table 2 and Figure S1. In men, the largest AUC was observed for the TyG index (AUC = 0.700, ES = 0.74, OR = 3.86, and optimal cut-off = 8.72). The prediabetes predictive values were similar for the TyG-WC (AUC = 0.689, ES = 0.69, OR = 3.53, and optimal cut-off = 844.20), TyG-BMI (AUC = 0.674, ES = 0.63, OR = 3.17, and optimal cut-off = 224.59), and TyG-WtHR (AUC = 0.667, ES = 0.61, OR = 3.02, and optimal cut-off = 5.27) indices. Conversely, the results for the ABSI AUC did not reach statistical significance (*p* = 0.066).

The largest AUCs for predicting prediabetes in women were observed for the TyG index (AUC = 0.695, ES = 0.72, OR = 3.79, and optimal cut-off = 8.92). The values for the AUCs for the TyG-WC (AUC = 0.654, ES = 0.56, OR = 2.76, and optimal cut-off = 802.81) and TyG-WHtR (AUC = 0.655, ES = 0.56, OR = 2.77, and optimal cut-off = 5.67) were similar, and presented acceptable values for predicting prediabetes in older Colombian women. Similarly, the AUC for the ABSI index did not reach statistical significance (*p* = 0.390).

### 3.5. Prevalence of Prediabetes According to Obesity- and Lipid-Related Indices

**Figure 9:**
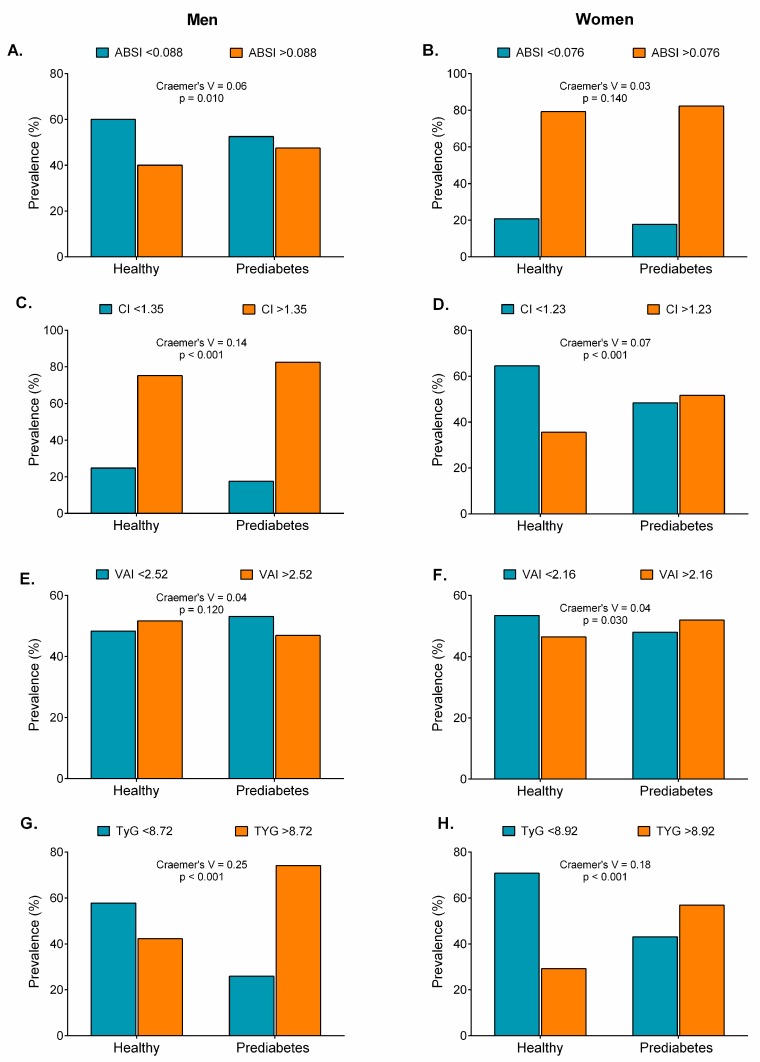
Prevalence of prediabetes according to obesity- and lipid-related index cut-offs (ABSI, C, VAI, and TYG).

**Figure 10:**
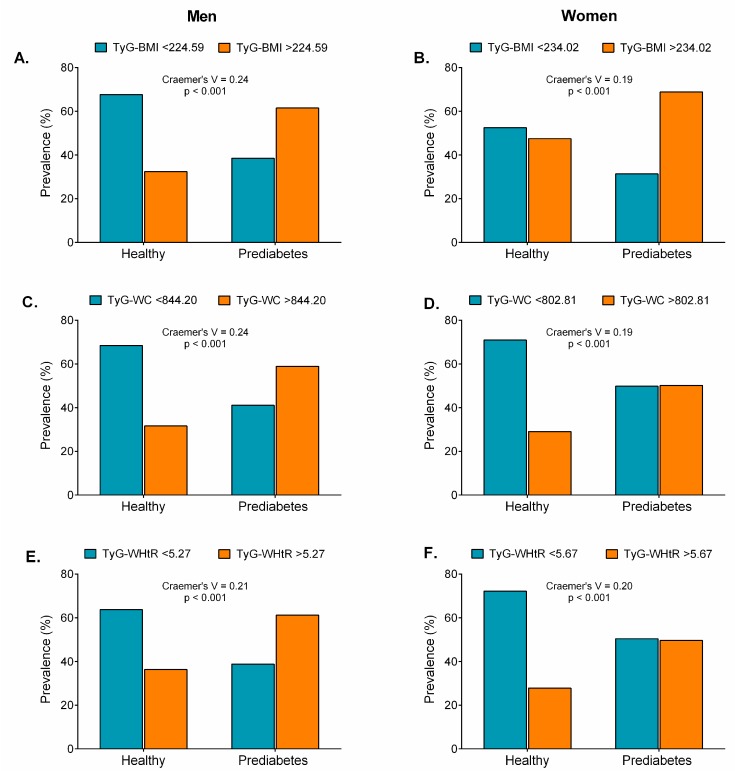
Prevalence of prediabetes according to obesity- and lipid-related index cut-offs (TyG-BMI, TyG-WC, and TyG-WHtR).

Compared with the healthy group, participants with prediabetes and high cut-off levels for obesity indicators had significantly higher BMIs, WCs, WHtRs, BRIs, Cs, VAIs, TyG indices, TyG-BMIs, TyG-WCs, and TyG-WHtRs (Figure 8, Figure 9 and Figure 10), except for the ABSI in women (Figure 9B) and the VAI in men (Figure 9E, *p* > 0.05). 

## 4. Discussion

This study evaluated the predictive ability of 11 obesity- and lipid-related parameters in identifying the risk of prediabetes in an elderly Colombian population, according to the ADA criteria published in 2016. This is an original study and, to the best of our knowledge, no report has previously compared the ability of these 11 surrogate clinical markers to indicate prediabetes in a large population of older adults.

Of the 3307 participants included, 839 (25.3%) were diagnosed with prediabetes. According to the International Diabetes Federation criteria, which are based only the IGT, the global prevalence of prediabetes in adults was estimated at 6.7% in 2015, and half of these individuals (50.1%) were aged under 50 years [29]. Based on glycated hemoglobin levels, the prevalence in the adult population in United Kingdom in 2011 was 35.5%, whereas in Spain, using the IFG or IGT alone, the incidence was 3.4% and 2.9% of the adult population, respectively, in 2010 [30]. In the USA, using the ADA definition and considering reference levels of glycated hemoglobin, IFG, or IGT, the prevalence of prediabetes was estimated at 38% in 2012 [31]. According to Vilanova et al., these differences justify the need for further research to establish a single definition for universally identifying prediabetic states [32].

In contrast, some anthropometric parameters measured in individuals with prediabetes in our study (BMI, WC, and the WtHR) were higher than those in healthy individuals. According to Barceló et al. [33] and Yang et al. [34], this finding could be explained by the fact that these parameters estimate patient adipose tissue content, which is correlated with prediabetic states. However, in agreement with Fujita et al. [35], who demonstrated that BMI, WC, and WtHR correlated better with prediabetes states than the ABSI index in 37,581 Japanese adults, our data indicate that the ABSI index did not correlate with prediabetes states.

Similarly, in a study of an American population followed up for more than 11 years, Hardy et al. demonstrated a poor correlation between ABSI and prediabetes states in adults, regardless of their race or sex [36]. Prediabetic adults also had higher serum triglyceride and glucose values than their non-prediabetic counterparts, which is consistent with the findings described by Yang et al. in a study with prediabetic Chinese adults [34]. Previous studies have reported that the VAI has significant advantages over WC or BMI measurements for determining cardiometabolic risk in young adults. However, in this study, the VAI was not a good surrogate marker for prediabetes and was an inadequate indicator in both men and women (AUCs = 0.564 and 0.575, respectively).

In agreement with previous studies [37,38,39], we found that hypertension and hearing loss were significantly higher among prediabetic adults. The development of comorbidities in prediabetic adults has become a point of concern for the scientific community, leading to continued research in the area in order to better understand the development of such complications, their treatment, and possible health-related consequences among prediabetic adults.

It has been suggested that obesity indices such as TyG index, TyG-BMI, TyG-WC, or TyG-WHtR values (involving the WC, BMI, TG, and HDL) might provide a broader evaluation of metabolic risk related to metabolic dysfunction and fat distribution. Thus, the TyG index has also recently been suggested as a method for estimating metabolic disorders. Indeed, in this study, the TyG index was more efficient compared to these other markers [32].

Our ROC analysis for prediabetes also highlighted differences according to sex. Thus, the TyG index combined with obesity indices was useful for predicting high glucose fasting levels. Among all the indices studied in men, the TyG and TyG-WC showed the highest OR for glucose levels (OR = 3.86, ES = 0.74 and OR = 3.53, ES = 0.69, respectively). Assuming that WC marks visceral adiposity, its combination with the TyG index better predicted prediabetes than other combinations.

In women, the highest ORs for prediabetes were observed by using the TyG index (OR = 3.79, ES = 0.72), followed by the TyG-WC (OR = 2.76, ES = 0.56). These results were consistent with the data reported by Lim et al., who concluded that the OR for prediabetes using the TyG-WtHR was higher than that with the TyG-BMI or TyG-WC for male Korean adults [23]. Unlike our results, these authors showed that TyG-WC produced the highest ORs for women.

In general, AUC = 1 indicates perfect predictive power, while AUC ≤ 0.55 indicates that the predictive power of an instrument is not better than chance. In this study, the TyG index cut-off points indicated sensitivity and specificity values between 69% and 70% for both sexes, thus moderately minimizing false-positive and false-negative cases. However, a very uncommon way of analyzing the diagnostic capacity of specific cut-off points is by calculating the positive (+LR) and negative (−LR) likelihood ratios.

In this study, the +LR was 1.74 in the male group and 1.90 in the female one, suggesting that male or female elderly individuals with a TyG index ≥8.72 or ≥8.92, respectively, have approximately twice the chance of having a positive diagnosis of prediabetes. The −LR was 0.43 and 0.58 in men and women, respectively, and was therefore also equivalent to twice the chance of a negative diagnosis of prediabetes in these populations, respectively. In this context, the TyG index is of greatest interest as a prediabetes predictive marker, because older adults at an increased risk of developing prediabetes or T2DM should be targeted by primary prevention efforts [40].

Similar to our study, Zheng et al. found that TyG-WC was the best marker for detecting prediabetes and diabetes [22]. Therefore, these results suggest that further studies of anthropometric markers related to the TyG index (with the potential to detect prediabetes among older adults) would be useful. These results contrast with those of Er et al., who concluded that the TyG-BMI index best predicts prediabetes in adults of either sex [40], putting the superiority of these obesity rate indices into doubt and further suggesting the need for additional studies on markers related to the TyG index.

Perhaps the disagreement in these studies on the ability of anthropometric indicators to predict prediabetes is the result of the ethnic origins of the populations used in these different studies [11,12,16,17]. The ethnicity of older adults can influence the definition of the cut-off points of the anthropometric indicators associated with glycemic status, which could impact their ability to identify a high risk of prediabetes. Thus, identification of the most appropriate anthropometric indicator and its respective cut-off points capable of predicting an increased risk of prediabetes may be dependent on the geographical location of each study. In addition, prediabetes can affect elderly patients for reasons other than excess fat and insulin resistance, including behavioral issues such as dietary intake, physical inactivity, sedentary lifestyles, sleep, and stress [41].

Finally, we must also mention the limitations and strengths of this study. First, its cross-sectional design limited our ability to interpret the associations we found. Second, because the sample population primarily comprised Colombians and some of the equations used to estimate obesity- and lipid-related parameters have not yet been validated in Hispanic/Latino populations, the generalizability of our results to other ethnic groups may be limited, and there may be some selective bias. However, these findings have important implications for public health policy targeted at improving access to care and chronic disease management for this rapidly growing population.

Our study also had several positive features. First, it included a community sample and allowed population estimates of the predictive ability of 11 obesity- and lipid-related parameters to identify patients with a high risk of prediabetes according to the 2016 ADA criteria to be established in an elderly Colombian population. Second, we also proposed optimal cut-off points for these anthropometric indices in clinical practice. Third, we studied older Colombian adults with diverse backgrounds and comorbid chronic diseases. Fourth, the large sample size and use of standardized methodological procedures in our study, as well as the fact that we developed this study within the SABE project framework to avoid measurement bias, were its strongest points.

Considering that recent studies on the TyG index have been extended to nonalcoholic fatty liver disease [42], coronary artery disease [43], insulin resistance [32,44], subclinical atherosclerosis [45], and T2DM [22], TyG-related markers deserve further study to identify their associations with the risk of prediabetes.

## 5. Conclusions

The TyG index best identified prediabetes in both sexes (AUCs and optimal cut-offs = 0.700 and 8.72 and 0.695 and 8.92 for men and women, respectively), suggesting that this index has the best discriminative power to predict prediabetes compared to other parameters, in both sexes. Thus, we propose TyG index as a complementary marker for assessing prediabetes in clinical practice and future epidemiologic studies among older adults. However, additional studies will be required to provide reference values applicable to different populations.

## Figures and Tables

**Table 1:** Clinical and sociodemographic characteristics of study participants according to glycemic status among Colombian older adults.

Variables	Total Sample (*n* = 3307)	Healthy (*n* = 2468)	Prediabetes (*n* = 839)	*p* for Groups
Age	69.8 (7.6)	69.7 (7.6)	70.2 (7.7)	0.331
**Anthropometric**				
Height (m)	1.56 (0.08)	1.56 (0.08)	1.55 (0.11)	0.143
Weight (kg)	65.1 (12.79)	63.84 (12.21)	68.1 (13.42)	<0.001
BMI (kg/m^2^)	26.78 (5.02)	26.32 (4.94)	28.13 (5.00)	<0.001
WC (cm)	92.20 (10.93)	91.1 (10.93)	95.41 (10.61)	<0.001
Waist height ratio	0.58 (0.09)	0.58 (0.09)	0.60 (0.08)	<0.001
Triglycerides (mg/dL)	159.55 (86.61)	153.43 (81.47)	175.43 (95.56)	<0.001
Glucose (mg/dL)	92.61 (11.61)	87.48 (8.43)	107.17 (6.49)	<0.001
**Obesity Indices**				
BRI	5.14 (2.02)	5.00 (1.97)	5.55 (2.10)	<0.001
ABSI (m^7/6^/kg^2/3^)	0.0803 (0.015)	0.0805 (0.014)	0.799 (0.017)	0.316
C (m^2/3^/kg^1/2^)	1.27 (0.24)	1.271 (0.23)	1.276 (0.27)	0.634
VAI	3.00 (3.16)	3.00 (3.15)	3.10 (3.19)	0.445
TyG index	8.78 (0.49)	8.70 (0.47)	9.03 (0.46)	<0.001
TyG-BMI	236.0 (48.90)	229.63 (47.25)	254.64 (48.92)	<0.001
TyG-WC	811.29 (116.65)	794.31 (111.94)	863.90 (112.58)	<0.001
TyG-WHtR	5.18 (0.91)	5.07 (0.91)	5.51 (0.82)	<0.001
**Weight Status**				
Underweight	78 (2.4)	66 (2.7)	12 (1.4)	0.305
Normal weight	1046 (31.6)	858 (34.8)	188 (22.4)	<0.001
Overweight	1299 (39.3)	943 (38.2)	356 (42.4)	0.209
Obesity	884 (26.7)	601 (24.4)	283 (33.7)	0.006
**Socioeconomic Status, *n* (%)**				
1 to 3 (Low)	3201 (96.8)	2388 (96.8)	813 (96.9)	0.917
4 to 6 (Medium to high)	106 (3.2)	80 (3.2)	26 (3.1)	0.536
**Smoking Status, *n* (%)**				
Yes	337 (10.2)	269 (10.9)	68 (8.1)	0.739
No	2970 (89.8)	2199 (89.1)	771 (91.8)	0.462
**Alcohol Intake, *n* (%)**				
Yes	418 (12.6)	326 (13.2)	92 (11.0)	0.739
No	2889 (87.4)	2142 (86.8)	747 (89.0)	0.043
**Physical Activity “proxy”, *n* (%)**				
Physically active	1503 (45.4)	1025 (41.5)	478 (57.0)	<0.001
Non-Physically active	1804 (54.6)	1443 (58.5)	361 (43.0)	<0.001
**Self-Report Comorbid Chronic Diseases, *n* (%)**				
Hypertension	1023 (30.9)	867 (35.1)	156 (18.6)	<0.001
Respiratory diseases	217 (6.6)	149 (6.0)	68 (8.1)	0.798
Cardiovascular diseases	311 (9.4)	219 (8.9)	92 (11.2)	0.737
Stroke	61 (1.8)	44 (1.8)	17 (2.0)	0.314
Osteoporosis	397 (12.0)	303 (12.3)	94 (11.2)	0.936
Cancer	109 (3.3)	84 (3.4)	25 (3.0)	0.590
Hearing loss	270 (8.2)	102 (4.1)	168 (20.0)	<0.001
Vision loss	919 (27.8)	700 (28.4)	219 (26.1)	0.622

Continuous variables are reported as mean values (standard deviations (SD) and categorical variables are reported as numbers and percentages in brackets. BMI: body mass index; WC: waist circumference; WHtR: waist to height ratio; BRI: body roundness index; ABSI: A body shape index; C: conicity index; VAI: visceral adiposity index; TyG: triglyceride and glucose index; TyGxBMI: TyG related to BMI; TyGxWC: TyG related to WC; TyGxWHtR: TyG related to WHtR.

**Table 2:** Cut-off between area under curve, sensitivity and specificity for obesity- and lipid-related indices to detect high prediabetes risk by sex.

Parameters	BMI	WC	WHtR	BRI	ABSI	C	VAI	TyG	TyG-BMI	TyG-WC	TyG-WHtR
	**Men**										
Area under curve	0.633	0.640	0.613	0.617	0.534	0.580	0.564	0.700	0.674	0.689	0.667
Effect Size	0.48	0.50	0.40	0.42	0.12	0.28	0.22	0.74	0.63	0.69	0.61
Odds Ratio	2.38	2.50	2.08	2.14	1.24	1.67	1.51	3.86	3.17	3.53	3.02
P-value	<0.001	<0.001	<0.001	<0.001	0.066	<0.001	<0.001	<0.001	<0.001	<0.001	<0.001
Optimal cutoffs	25.58	96.0	0.57	4.96	0.088	1.35	2.52	8.72	224.59	844.20	5.27
J-Youden	0.23	0.21	0.19	0.20	0.080	0.14	0.12	0.32	0.30	0.29	0.28
Sensitivity (%)	62.10	59.10	61.21	58.43	21.87	56.10	60.89	75.63	68.04	61.26	55.18
Specificity (%)	60.93	62.18	58.57	62.45	86.36	58.49	51.95	57.05	62.19	68.63	73.56
(+) Likelihood ratio	1.59	1.56	1.57	1.56	1.60	1.35	1.27	1.74	1.80	1.95	2.09
(−) Likelihood ratio	0.62	0.56	0.68	0.67	0.90	0.75	0.75	0.43	0.52	0.56	0.61
	**Women**										
Area under curve	0.603	0.597	0.600	0.596	0.504	0.573	0.575	0.695	0.642	0.654	0.655
Effect Size	0.36	0.34	0.35	0.34	0.01	0.26	0.26	0.72	0.51	0.56	0.56
Odds Ratio	1.95	1.87	1.91	1.86	1.02	1.60	1.62	3.79	2.54	2.76	2.77
P-value	<0.001	<0.001	<0.001	<0.001	0.390	<0.001	<0.001	<0.001	<0.001	<0.001	<0.001
Optimal cutoffs	26.57	90.0	0.60	5.51	0.076	1.23	2.16	8.92	234.02	802.81	5.67
J-Youden	0.17	0.17	0.16	0.17	0.04	0.13	0.13	0.28	0.23	0.25	0.23
Sensitivity (%)	70.36	65.61	61.01	61.45	84.31	63.45	67.22	60.77	75.81	70.38	51.57
Specificity (%)	47.49	51.80	55.43	55.60	19.80	49.97	45.93	68.08	47.80	54.63	71.47
(+) Likelihood ratio	1.34	1.36	1.37	1.38	1.05	1.27	1.24	1.90	1.44	1.55	1.80
(−) Likelihood ratio	0.62	0.66	0.70	0.69	0.79	0.73	0.71	0.58	0.51	0.54	0.68

BMI: body mass index; WC: waist circumference; WHtR: waist to height ratio; BRI: body roundness index; ABSI: A body shape index; C: conicity index; VAI: visceral adiposity index; TyG: triglyceride and glucose index; TyG-BMI: TyG related to BMI; TyG-WC: TyG related to WC; TyG-WHtR: TyG related to WHtR.

## Supplementary Materials

The following are available online at https://www.mdpi.com/2072-6643/11/11/2654/s1, Figure S1: Diagnostic properties of ratio of obesity- and lipid-related indices to detect high risk of the prediabetes according to the American Diabetes Association in 2016 by sex.
