# Supplementary material for: Obesity- and Lipid-Related Parameters in the Identification of Older Adults with a High Risk of Prediabetes According to the American Diabetes Association: An Analysis of the 2015 Health, Well-Being, and Aging Study
Source: Nutrients. 2019 Nov 4;11(11):2654. doi: 10.3390/nu11112654 (PMC6893527; doi:10.3390/nu11112654)

Supplementary Materials:

Figure S1: Diagnostic properties of ratio of obesity and lipid-related indices to detect high risk of the prediabetes according to the American Diabetes Association in 2016 by sex.

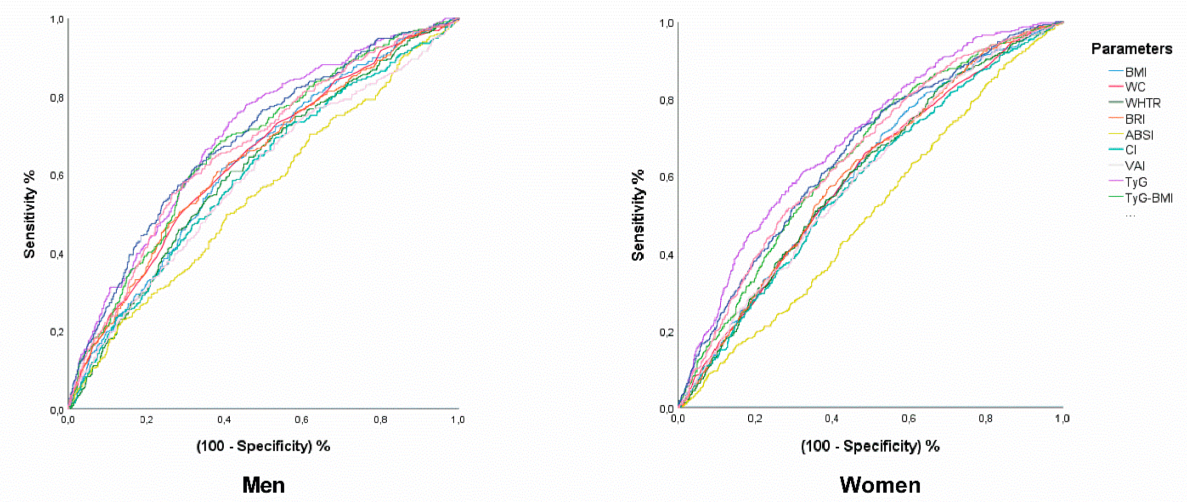

Supplement: Supplementary file 1 [file nutrients-11-02654-s001.pdf]
